# Peer review: a collective commitment to knowledge and excellence

**DOI:** 10.1093/braincomms/fcaf498

**Published:** 2026-01-09

**Authors:** David Belin, Tara L Spires-Jones

**Affiliations:** Cambridge, UK; Edinburgh, UK

## Abstract

Our editors discuss the importance of fair and constructive peer review while extending thanks to all those who have contributed their expertise in reviewing manuscripts for *Brain Communications.*

The landscape of scientific communication has been evolving faster than ever over the past years. Post-publication peer review and preprint platforms such as F1000 and bioRxiv, respectively, which were both launched in 2013 and have since been joined by several others, including the preprint service for medical research medRxiv, have transformed how research is disseminated, allowing scientists to share their findings with unprecedented speed and reach while receiving live feedback from the community. Preprints have become invaluable tools for transparency, collaboration and early access to knowledge—particularly in times of urgent global challenges. Yet, as the pace of publication accelerates, it becomes ever more vital to reflect on the enduring role of peer-reviewed scientific journals and the peer-review process: not as a gatekeeping ritual but as a collaborative endeavour that should help authors publish the best, most impactful version of their work.

At its best, peer review is an act of intellectual generosity and an opportunity to learn for all parties involved. It represents a dialogue among peers with shared expertise, committed to advancing collective understanding, rather than individual prestige. Reviewers bring to a manuscript not only their technical expertise but also the perspective of a thoughtful reader who can identify logical gaps, methodological weaknesses or overextended claims that authors—understandably immersed in their data—may overlook. Far from being a punitive exercise, the process should really help refine and strengthen a study, enhancing its clarity, interpretability and reproducibility, if necessary. Unfortunately, the system is too often used to demean, bully and/or prevent publication of results that may not align with the prevailing view of the time or with the reviewer’s opinion. Too often, we, as authors, are asked to perform additional experiments that would only satisfy a reviewer’s curiosity, rather than directly addressing the question at hand.

Such practice, which has no place at *Brain Communications*, has resulted in a complete erosion of the dialogical nature of peer review, which should otherwise foster a culture of trust—both within the scientific community and between science and society. Editors, who rely on reviewers not simply as judges but as partners who ensure that decisions are fair, transparent and informed, play a very important role in ensuring the peer-review process of their journal delivers what it was originally designed to do. Such mutual trust between authors, reviewers and editors is the foundation upon which scientific scholarship is built. We strongly believe that such trust can be achieved only among peers, particularly in society journals.

However, maintaining this trust when it has not yet been completely eroded demands constant ongoing adaptation and pedagogical work on the part of the editors, whose job is also to help reviewers hone their craft. The coexistence of preprint dissemination and traditional peer review challenges us to rethink what added value a peer-reviewed journal can offer. Ultimately, the peer-review process thrives when it is viewed not as a transaction but as a shared responsibility. Authors who engage constructively in a dialogue based on balanced and constructive critique from reviewers, and editors who mediate with fairness, all contribute to the integrity of the scientific enterprise, which should be intellectually fulfilling for all parties. In this sense, peer review is more than a mechanism of quality control; it is a social contract that enables us to publish the best version of our research.

The challenge for the next decade is to reimagine peer review in ways that preserve its strengths while aligning it with the openness and immediacy that characterize modern science. By engaging in review with curiosity and care, we affirm our collective commitment to the scientific endeavour and to each other.

At *Brain Communications*, this commitment is reflected daily in the thoughtful, detailed and generous reviews provided by our community. Seldom have we, the editors, had to engage with a reviewer on the content of their review, which speaks to the mindset of our community. Our Reviewer Academy initiative ensures that the next generation of reviewers trained by *Brain Communications* will share this mindset and the values that underpin constructive, fair peer review.

Each review represents an investment of time and expertise that strengthens the work we publish and supports the authors behind it. As editors, we are deeply grateful for this dedication. The continued growth of *Brain Communications* rests upon the integrity, insight and collegial spirit of our reviewers.

We therefore take this opportunity to extend our heartfelt thanks to all those who have contributed their time and expertise since the launch of the B*rain Communications* almost 7 years ago. We are particularly grateful to Professor David Brooks, Professor Nicola Pavese and Dr Niels P. Bergsland, who have each reviewed over 25 manuscripts for *Brain Communications*, as well as to Drs Yuto Uchida and Lena Oestreich for reviewing over seven manuscripts for our journal last year.

Your efforts exemplify the best of scientific citizenship—advancing not only individual manuscripts but also the collective enterprise of neuroscience. We look forward to continuing this shared journey towards excellence, transparency and trust.

The cover image for this issue comes from Reyes-Pablo *et al*.^1^ and shows a layer II entorhinal cortex lacuna containing neurofibrillary tangles labelled with tau markers, illustrating the pathological accumulation characteristic of neurodegenerative disease.
